# Cuboid fracture: An extremely rare cause of limping in a 2‐year‐old child

**DOI:** 10.1002/ccr3.6021

**Published:** 2022-07-11

**Authors:** Ioannis Delniotis, Georgios Nitis, Christos Gekas, Benedikt Leidinger

**Affiliations:** ^1^ Department of Orthopedics and Pediatric Orthopedics Hippokration General Hospital Thessaloniki Greece; ^2^ Department of Orthopaedics Kavala General Hospital Kavala Greece; ^3^ Department of Pediatric Orthopedics, Neuro‐Orthopedics, Foot & Ankle Surgery Orthopaedic Hospital Volmarstein Wetter Germany

**Keywords:** child, cuboid, fracture, limping

## Abstract

The purpose of this case report is to raise awareness of an extremely rare cause of limping, in young children. Clinicians should have a high index of suspicion of a possible cuboid fracture when evaluating a young child who presents with limping.

## Graphical abstract

Cuboid fracture is a rare cause of limping, in young children. Clinicians should have a high index of suspicion of a possible cuboid fracture in young children who refuse to bear weight on the lateral side of the foot, even without a trauma history.

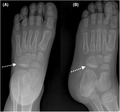

## 1 CASE DESCRIPTION

A 2‐year‐old girl presented to our clinic with a 3‐week history of limping and no signs of pain. A recent upper respiratory infection but no other associated systemic or genetic problems are mentioned. No history of trauma is reported, except for a possible minor injury. Two prior evaluations of the child were performed. Blood tests and bilateral hip ultrasound were performed. All values were normal, and no hip effusion was found. Range of motion of the lower extremities was normal. Despite minimizing walking, the child continued to limp.

**FIGURE 1:**
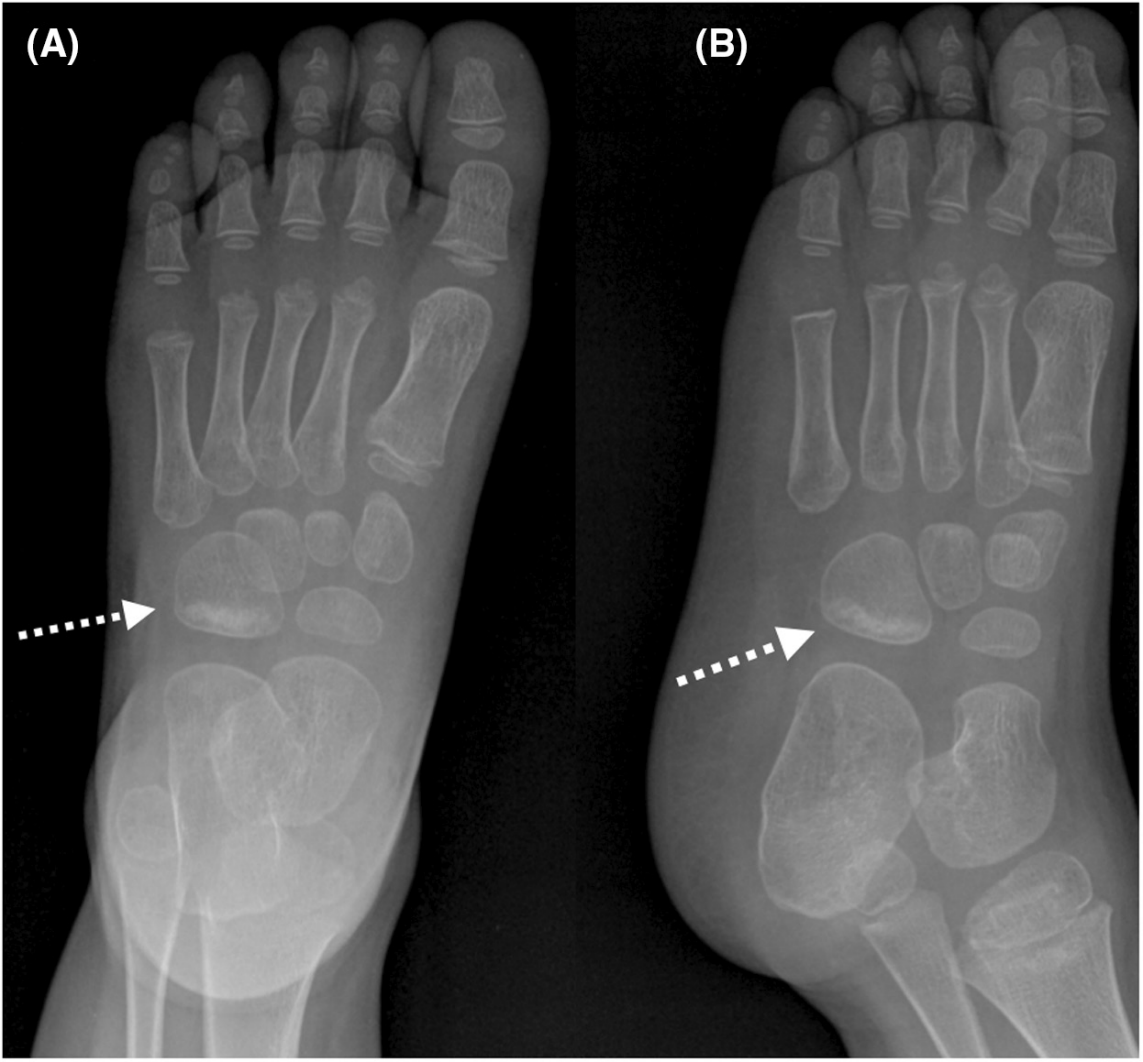
(A + B): F + profile view of the left foot, 3 weeks after limping. The dotted arrows show proximal callus formation of the cuboid bone, thus revealing the cause of limping (cuboid fracture)

When the child came to our department, we examined thoroughly the gait pattern. We noticed that the child refused to bear weight on the lateral side of the left foot. Palpation of the left foot did not reveal any tenderness but the “nutcracker” maneuver was postive.^1^ Plain radiographs of the left foot revealed the fracture (Figure 1). A below‐knee cast was applied for 3 weeks.

A limping child can be a difficult diagnostic challenge for clinicians.^2^ Doctors should have a high index of suspicion of a cuboid fracture in young children who refuse to bear weight on the lateral side of the foot, even without a significant trauma history.

## AUTHOR CONTRIBUTIONS

ID drafted the manuscript and contributed to patient care. GN revised the manuscript. CG contributed to patient care. BL revised the manuscript and final approval of the manuscript.

## CONFLICT OF INTEREST

There are no conflicts of interest associated with this publication, and there has been no financial support for this work that could have influenced its outcome.

## CONSENT

Written informed consent to publish this report was obtained from the family before the submission process. Written informed consent was obtained from the patient to publish this report in accordance with the journal's patient consent policy

## Data Availability

The data that support the findings of this study are available from the corresponding author upon reasonable request. The data are not publicly available due to privacy or ethical restrictions.
